# Tai chi, irisin and cognitive performance: a clinical and biological investigation in older adults

**DOI:** 10.1007/s40520-024-02743-5

**Published:** 2024-04-10

**Authors:** Anna Giulia Guazzarini, Francesca Mancinetti, Patrizia Bastiani, Michela Scamosci, Roberta Cecchetti, Virginia Boccardi, Patrizia Mecocci

**Affiliations:** 1 Division of Gerontology and Geriatrics, Department of Medicine and Surgery, University of Perugia, Santa Maria della Misericordia Hospital, Piazzale Gambuli 1, Perugia, 06132 Italy; 2 Division of Clinical Geriatrics, NVS Department, Karolinska Institutet, Stockholm, Sweden

**Keywords:** Aging, Cognition, Irisin, Tai Chi, Prevention

## Abstract

**Background:**

Skeletal muscle is the main source of circulating irisin, both at rest and during physical activity. Previous studies have suggested that irisin can improve cognitive abilities.

**Aims:**

We explored whether six months of Tai Chi (TC) practice can modulate such a relationship in healthy older persons.

**Methods:**

This is a prospective clinical study to evaluate the effects of TC practice as compared with low intensity exercise (LI) and no exercise (NE) control groups on plasmatic irisin levels and cognitive performance. Forty-two healthy older persons were stratified into three groups according to physical activities. Biochemical assay and cognitive functions were assessed at the baseline and after six months.

**Results:**

A significant change was found in circulating irisin levels in TC as compared with NE group (*p* = 0.050) across time. At six months in TC group irisin levels significantly correlated with a verbal memory test (*p* = 0.013) controlled by age and education.

**Conclusion:**

Our results suggest the potential benefits for cognitive health of TC practice by irisin levels modulation.

## Introduction

Age is a key determinant of cognitive decline and dementia, with its prevalence rising significantly as individuals grow older [1]. Nevertheless, the aging process varies among individuals, and an expanding body of literature indicates a correlation between physical exercise and trajectories of aging, as well as outcomes related to dementia [2]. Considering there is no cure for dementia, non-pharmacological approaches in disease prevention by diet and physical activity are of great interest. Several types of exercise programs have been described, and every kind of intervention has its own characteristics and expected effects on skeletal muscle and function, and on other body systems, including brain and related cognitive function [3].

Among the various exercise activities, Tai Chi is considered a good option for older adults [4]. Previous studies have shown that it can improve aerobic capacity, muscle strength, balance, and motor control, as well as induce a reduction in stress and anxiety in older persons [4]. Accordingly, a more recent review reported that the effects of Tai Chi on neurocognitive outcomes in people with initial cognitive decline and early-stage dementia are still inconclusive, calling for further clinical studies to understand if and how Tai Chi can be applied as an effective intervention to delay cognitive impairment [5]. Mechanisms by which Tai Chi can influence cognitive performances are still unknown and under investigation.

Physical activity exerts a positive regulatory influence on every organ within the body, employing diverse mechanisms to achieve this effect. In this context, myokines are an extensive group of proteins, peptides, and metabolites produced by muscle with endocrine activity. Irisin is largely released from skeletal muscle after exercise and, to a lesser extent, from white adipose tissue [6]. Originally, its actions included increased energy expenditure, improved insulin sensitivity, and induced weight loss; it also appeared to stimulate lipolysis and inhibit lipid accumulation in adipocytes [7]. Interestingly, recent clinical studies have shown that irisin not only influences muscle tissue but also plays a role in synaptic regulation in the brain [8]. Notably, its impact on the nervous system is noteworthy, exhibiting a remarkable capacity to enhance cognitive function. Thus, a link between Tai Chi practice, irisin, and brain functions cannot be ruled out. No study so far has evaluated the effect of Tai Chi practice over a long period of time on cognitive domains revealed by a comprehensive assessment. Considering such evidence, we sought to examine the impact of practicing Tai Chi on cognitively healthy and older subjects aged 55–89 years, to assess any changes in plasma irisin level as well as in cognitive performances.

## Methods

### Study design

This is a single- center, open-label, 3-arm clinical study, conducted for a total duration of six months. This study compared the effects of practicing Tai Chi (TC) with those of low intensity exercise (LI) and no exercise (NE) control groups on physical and cognitive performance in healthy older subjects.

We enrolled subjects of both sexes and aged between 55 and 89 years, cognitively intact (MMSE score > 26) and referred to the outpatient service of the Section of Gerontology and Geriatrics of the Department of Medicine and Surgery of the University of Perugia and to members of the “Pian di Massiano” Senior Citizens Center of Perugia. Subjects with ongoing malignancies or diagnosed within five years, with active infections or diseases of autoimmune pathogenesis, with unintentional weight loss > 5 kg in the last 12 months, with stage III chronic renal insufficiency, with psychiatric disorders and diseases limiting motor and cognitive abilities or unable to sign informed consent were excluded. A total of 42 healthy persons were includeded and provided informed consent. The study adhered to the Declaration of Helsinki and was approved by the Regional Ethics Committee (N. 24,444/22/ESS).

### Physical exercise and group definition

The term “exercise” refers to a systematically structured regimen of physical activity, while “exercise intensity” denotes the level of exertion experienced during physical activity. Typically, low intensity exercise for older adults encompass activities such as leisurely walking, gentle stretching, utilizing light hand weights, performing sit-ups, and engaging in wall-assisted push-ups. Programs tailored for older adults often incorporate combination exercises with low intensity. Tai Chi, originating in seventeenth-century China, represents a traditional mind-body exercise characterized by its integration of physical, cognitive, and meditative elements into a unified practice. Renowned for its gentle demands, Tai Chi offers both physical and mental conditioning, making it an ideal choice for older adults seeking comprehensive wellness benefits.

Following enrollment, participants were randomly allocated to distinct exercise groups. The NE group served as the control, receiving general advice on maintaining a healthy lifestyle. Participants in the TC group engaged in Tai Chi, attending two sessions per week. Meanwhile, those in the LI group underwent low intensity exercises, involving two weekly sessions integrating both aerobic workouts and strength training. All subjects underwent baseline assessments, followed by a re-evaluation after six months. During the interim period, participants assigned to the TC and LI groups actively participated in association activities, whereas those in the NE group adhered solely to the scheduled protocol visits.

### Clinical and biochemical assessment

Clinical information was obtained by means of a medical history and routine laboratory analysis. Family (including neurocognitive impairment), remote pathological and pharmacological history were collected through self-reported information from the participants. Anthropometric determinations (weight, height, waist, and hip circumference) were measured using standard techniques. Body mass index (BMI) was calculated as weight in kilograms divided by the square of height expressed in meters (kg/m^2^).

Blood samples were collected in the morning after the participants had been fasting for at least 8 h. Blood glucose, creatinine, cholesterol, and triglycerides were analysed using enzymatic methods, whereas high-density lipoprotein (HDL)-cholesterol was measured after isolation of low-density lipoprotein (LDL) and very-low-density lipoprotein (VLDL; Boehringer Mannheim GmbH, Germany) and LDL cholesterol was calculated using Friedewald’s method.

### Cognitive assessment

Neuropsychological assessment was performed as extensively previously reported [9]. Cognitive performance was assessed during the screening visit with the Mini-Mental State Examination (MMSE) and the Addenbrooke’s Cognitive Examination-Revised (ACE-R) a neuropsychological battery explores five cognitive domains (orientation, memory, fluency, language, and visual-spatial function) as a test of screening and general cognition. All subjects then underwent a large battery of neuropsychological tests aimed at assessing the different cognitive areas including memory by the Digit Span forwards and backwards and the Rey’s Auditory Verbal Learning Test (RAVLT I, immediate and RAVLT D, delayed recall); attention by the Trail Making test part A (TMT A) and executive function by the Trail Making test part B (TMT B).

### Plasma irisin measurement

The determination of plasma irisin was carried out on plasma and analyzed in duplicate by the ELISA method. At the recruitment, blood samples were collected in EDTA tubes from a peripheral vein after overnight fasting and kept immediately on ice. Plasma was separated by centrifugation (4000 rpm for 15 min at 4 °C), aliquoted, and stored at − 80 °C until analyzed. The analytes concentration was calculated using a standard curve, with software provided by the manufacturer (Bio-Plex Manager Software).

### Statistical analyses

The observed data were normally distributed (Shapiro–Wilk W-Test) and are presented as means ± Standard Deviation (SD). Chi-squared (χ^2^) test, simple and partial Pearson correlations, mixed repeated measure by analyses of variances (ANOVA) followed by Tukey’s post hoc test, ANOVA followed by Fisher’s least significant difference (LSD) procedure were used as appropriate. Delta values for irisin were also calculated as: (Delta = irisin at six months − irisin at baseline). All *p* values are 2-tailed, and the level of significance was set at *p* ≤ 0.05. Statistical analyses were performed using the SPSS 22 software package (SPSS, Inc., Chicago, IL, USA).

## Results

### Sample characteristics

The study evaluated 42 subjects with no difference in gender distribution, slightly overweight (BMI: 26.71 ± 4.00 Kg/m^2^), and with a mean age of 70.64 ± 5.81 years. The clinical and biochemical characteristics of the study population at baseline and stratified for the three randomization groups are shown in Table 1. Subjects in the TC group had significantly higher education levels than other groups. No significant differences were found in the anthropometric measurements of weight, height, and measure of waist and hip circumference, as well as in the results of the glycometabolic and lipidic profiles. Repeated measured by ANOVA revealed a significant difference in total cholesterol changes over time, showing a reduction in NE and LI group (F:4.829, *p* = 0.034). No other differences in all other anthropometric and biochemical variables examined after six months were found.

**Table 1:** Basal clinical and biochemical characteristics of the population sample (*n* = 42)

	Total sample *n* = 42	NE *n* = 14	TC *n* = 14	LI *n* = 14	*p*-values
**Gender (F/M, n)**	28/14	8/6	10/4	10/4	0.651*
**Age (years)**	70.64 ± 5.81	71.92 ± 5.22	69.57 ± 6.98	70.42 ± 5.25	0.566
**Education (years)**	13.14 ± 3.88	11.93 ± 4.17	15.43 ± 2.84	12.07 ± 3.66	0.022
**Weight (kg)**	70.83 ± 13.15	72.93 ± 14.54	71.14 ± 11.83	68.43 ± 13.51	0.671
**Height (cm)**	162.55 ± 9.32	162.71 ± 11.69	164.14 ± 6.98	160.79 ± 9.21	0.644
**BMI (kg/m**^**2**^)	26.71 ± 4.00	27.17 ± 3.64	26.56 ± 4.45	26.41 ± 4.14	0.872
**Waist circumference (cm)**	88.74 ± 11.64	88.86 ± 11.68	88.00 ± 12.50	89.36 ± 11.58	0.955
**Hip circumference (cm)**	106.00 ± 12.83	104.29 ± 8.33	110.00 ± 19.20	103.71 ± 7.40	0.367
**Waist/Hip ratio**	0.84 ± 0.10	0.85 ± 0.09	0.81 ± 0.14	0.86 ± 0.08	0.546
**Glycemia (mg/dl)**	98.72 ± 17.75	102.86 ± 20.21	98.07 ± 17.75	94.93 ± 19.04	0.504
**Creatinine (mg/dl)**	0.81 ± 0.17	0.78 ± 0.20	0.79 ± 0.20	0.86 ± 0.08	0.417
**Total Cholesterol (mg/dl)**	209.64 ± 50.36	219.57 ± 38.74	189.42 ± 72.68	219.92 ± 23.60	0.186
**LDL-Cholesterol (mg/dl)**	132.27 ± 35.91	135.78 ± 34.13	121.35 ± 45.56	139.67 ± 25.04	0.373
**HDL-Cholesterol (mg/dl)**	62.38 ± 14.88	61.79 ± 15.44	63 ± 15.04	62.21 ± 15.25	0.971
**Triglycerides (mg/dl)**	96.88 ± 37.83	110.00 ± 42.49	92.36 ± 33.33	88.29 ± 36.19	0.278

NE: no exercise; LI: low intensity exercise; TC: Tai Chi; BMI: Body mass index. *χ^2^ = 0.857

### Irisin evaluation and cognitive assessment

**Fig. 1:**
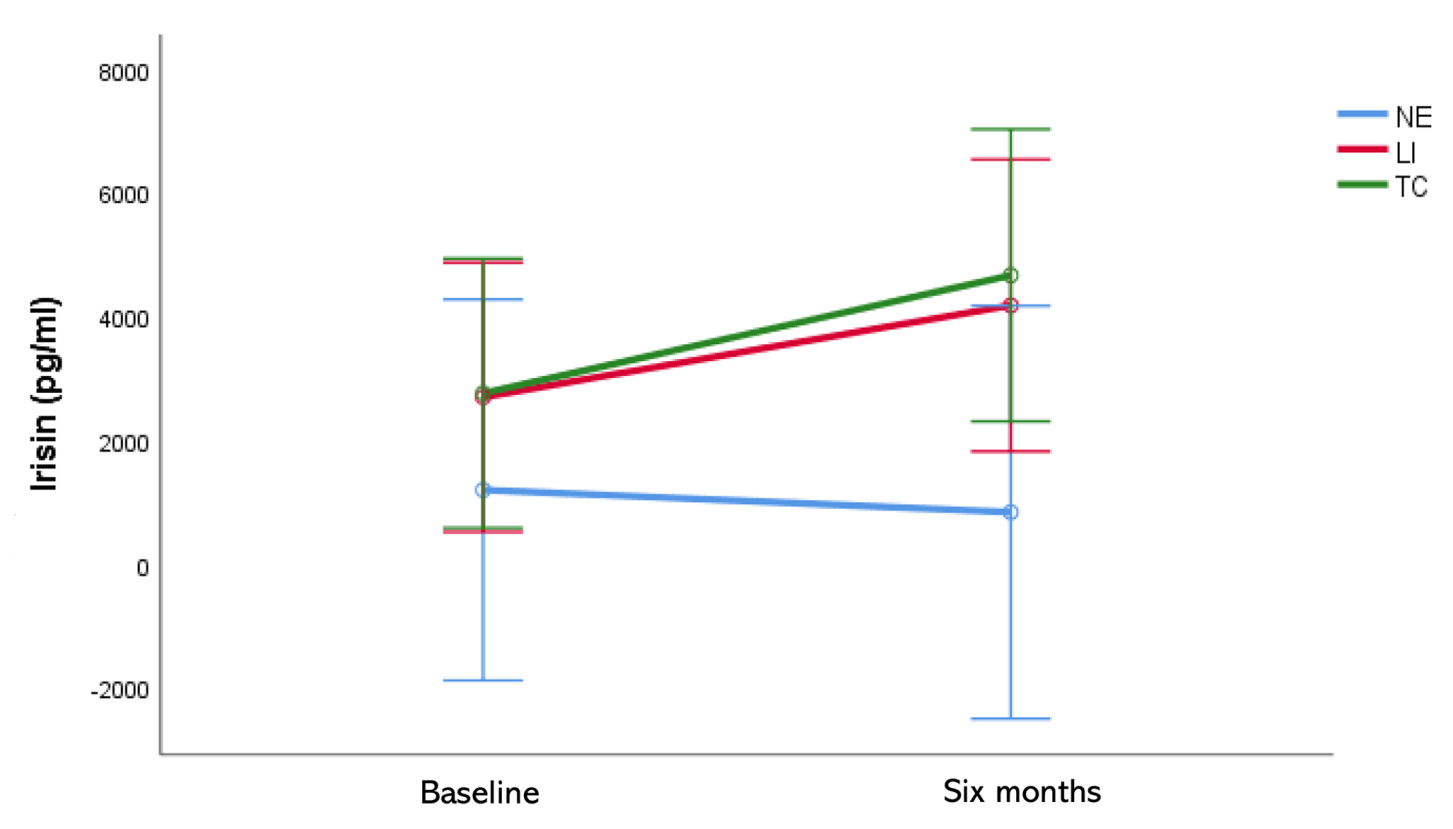
Plasmatic irisin levels changes across time (from baseline to six months) between independent groups of physical exercise NE: no exercise; LI: low intensity exercise; TC: Tai Chi. F = 9.338, *p* = 0.005 within subjects over time by mixed ANOVA for repeated measurement

**Fig. 2:**
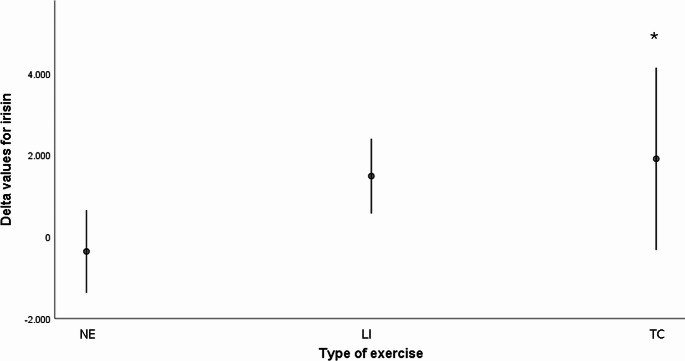
Changes in plasmatic irisin levels among groups of physical exercise NE: no exercise; LI: low intensity exercise; TC: Tai-Chi * *p* = 0.050 TC vs. NE. LI vs. NE *p* = 0.145; TC vs. LI *p* = 0.677. All *p* values we obtained using ANOVA followed by post hoc Fisher’s least significant difference (LSD)

No difference was found in irisin levels at baseline and at six months between gender (*p* = 0.448) in all population, as well as no correlation was found between irisin levels and age at baseline (*r* = 0.101, *p* = 0.565) and at six months (*r* = 0.144, *p* = 0.415). A mixed model ANOVA was performed to compare irisin levels change across time between the independent groups of physical exercise and reported in Fig. 1. A significant change in irisin levels within time (F:4.571, *p* = 0.042) was found: an increase in irisin levels in TC and LI groups from baseline (TC: 2757.47 ± 3229.88 pg/ml; LI: 2693.16 ± 4627.35) to six months (TC: 4662.30 ± 4863.54 pg/ml; LI: 4176.09 ± 4321.32) and a slow reduction in NE (baseline: 1193.93 ± 1118.50; six months: 834.47 ± 497.99), even if the difference between groups across time did not reach the statistical significance (F = 1.159, *p* = 0.329). The post hoc analysis, instead, revealed a trend for difference between TC vs. NE groups (*p* = 0.077) across time. To better quantify the measured changes Delta values for irisin were also calculated. A significant change in irisin levels over six months was found between NE and TC (-659.46 ± 961.78 vs. 1904.82 ± 3506.47, *p* = 0.050) groups. No difference was found between NE and LI (1482.93 ± 1434.86, *p* = 0.145) as well as between LI and TC (*p* = 0.677) groups (Fig. 2). To test whether the relative baseline and after six months irisin values were related to cognitive performances, multiple Pearson’s correlation tests were performed. No correlation was found among all cognitive variables examined and irisin at baseline among groups (data not shown). At six months only in TC group irisin levels significantly correlated with RALVT D score (*r* = 0.715, *p* = 0.013) while a trend was found in ACE-R total (*r* = 0.553, *p* = 0.078) and TMT A (*r*=-0.576, *p* = 0.064) controlled by age and education. No other difference was found.

## Discussion

Collectively our study demonstrates that: (1) plasma irisin levels increase and change significantly in the Tai Chi group compared to the group performing no physical activity over six months, (2) at six months in subjects in TC groups irisin levels correlated significantly with the Auditory Verbal Learning Test delayed (RALVT D) as a test of memory function.

Tai Chi, an ancient meditative martial art with roots in China spanning centuries, has garnered growing popularity in Western societies. This practice encompasses a sequence of serene movements designed to fortify and soothe both the body and mind. Although various schools of Tai Chi exist, they collectively emphasize fundamental principles such as mindfulness, structural alignment, and flexibility, contributing to its universal appeal and holistic benefits [10]. The effects of Tai Chi are diverse and can positively impact different aspects of cognitive well-being. A recent meta-analysis showed that subjects who practiced Tai Chi had better Mini-Mental Status Examination scores and depression assessments when comparing the Tai Chi cohort with the non-exercise control cohort rather than the exercise control cohort [11]. While research on the specific biological mechanisms of Tai Chi in cognitive performance is still ongoing, no study so far has evaluated the potential connection between Tai Chi and irisin.

Myokines represent a category of molecules with pluripotent effects, including cytokines and signaling peptides which are expressed, synthesized, and released by skeletal myocytes in response to muscle contractions [12]. Irisin is a myokine that is produced by the body during physical activity, especially during aerobic exercise [12], well known for its potential role in metabolism and the regulation of energy expenditure. Some studies suggest that it may induce the browning of white adipose tissue, converting it into a more metabolically active form that can help burn calories and regulate body weight [13]. It has been previously shown the impact of irisin gene deletion on cognitive function in mouse model of Alzheimer’s disease (AD) [14]. Moreover, their study revealed that administering irisin peripherally in two distinct AD mouse models facilitated its passage across the blood-brain barrier, thereby mitigating cognitive decline associated with pathological alterations. Kuster and colleagues found [15] a strong connection between irisin levels and both episodic memory and global cognition among individuals at risk of dementia. Similarly, another investigation uncovered a favorable relationship between irisin concentrations and neurophysiological performance in obese individuals at risk of AD [16]. Moreover, they suggested a potential link between plasma irisin levels and neurocognitive impairments observed during visuospatial working memory tasks.

In our study involving a cohort of older healthy subjects, we observed that engaging in Tai Chi for a duration of six months was correlated with a significant elevation in plasma irisin levels. After six months irisin levels significantly correlated with Rey’s Auditory Verbal Learning Test (RALVT) Delayed score, controlled by age and education. RAVLT is a neuropsychological assessment tool used to evaluate an individual’s long term verbal memory and learning abilities. The function of memory is an important aspect of the brain, responsible for retaining and organizing acquired knowledge, allowing individuals to adapt their behavior based on lifelong experiences. Deficiencies in memory recall can significantly impact an individual’s daily activities and overall well-being. Thus, the potential improvement of memory function by Tai Chi practice and potentially mediated by irisin, holds significance in the context of reducing the risk of cognitive decline and sustaining cognitive function. Collectively, it is possible to hypothesize that the strong correlation between Tai Chi and psychophysical well-being with protection in neurocognitive processes is related to the activity that the muscle performs through a muscle-brain connection [17].

However, the secretion of irisin and its protective effect on the nervous system is a complex process involving multiple factors. Many research findings are contradictory, which can be attributed to a variety of factors such as the selection of time points for irisin measurement after exercise, cleavage of irisin during storage, detection kit accuracy, and so on. Our study has several strengths, including the use of randomization as a major distinguishing feature of this study design. However, some limitations need to be reported as the small simple size. Thus, more extensive studies are necessary to validate our data and better clarify the network in which irisin are involved. In conclusion while the specific mechanisms linking Tai Chi, irisin, and memory function are still being elucidated, the multifaceted nature of Tai Chi suggest that its potential benefits for cognitive health may involve a combination of physical, mental, and physiological factors. Tai Chi may represent an affordable and effective practice to improve cognitive function, particularly in older persons who are most vulnerable to cognitive decline and neurodegenerative disorders. Ongoing research in this area will likely provide further insights into the role of Tai Chi and related hormones in supporting cognitive function across the lifespan.

## Data Availability

No datasets were generated or analysed during the current study.
